# Origins of Atlantic Salmon (*Salmo salar*) Determined Using a Hybridization Assay of Mitochondrial DNA on a Microfluidic Biochip

**DOI:** 10.3390/bios16040231

**Published:** 2026-04-21

**Authors:** Lin Wang, Christopher Oberc, Krzysztof P. Lubieniecki, William S. Davidson, Paul C. H. Li

**Affiliations:** 1 Department of Chemistry, Simon Fraser University, 8888 University Drive, Burnaby, BC V5A 1S6, Canada; 2 Department of Molecular Biology and Biochemistry, Simon Fraser University, 8888 University Drive, Burnaby, BC V5A 1S6, Canada

**Keywords:** microfluidic biochip, Atlantic salmon, mitochondrial DNA, hybridization assay, single nucleotide polymorphism

## Abstract

A hybridization assay based on the microfluidic biochip was developed to identify the origin of the Atlantic salmon species. Among the 215 single nucleotide polymorphic (SNP) sites found in the mtDNA of *Salmo salar*, we located five sites in devising our assay method. We found two sites that worked, while the others generated either insufficient signals or specificity. We have successfully identified the North American origin of the three samples, as confirmed by Sanger sequencing.

## 1. Introduction

Atlantic salmon (*Salmo salar*) has routinely been identified for its continental origin, viz., North American or European [1]. Commercially, salmon stocks are harvested together at sea when they are mixed on feeding grounds or migration routes [2,3]. The proportions of continent of origin vary annually or even seasonally [1,4]. For example, the proportion of caught salmon of North American origin has more than doubled since the 1970s [1]. Mixed-stock fishery has raised a lot of concerns because it can cause less productive stocks to be seriously depleted and more productive ones under-harvested [5,6]. This situation implies a genetic risk to salmon stocks. Therefore, information on the composition and origin of the catch should be provided to management for setting up the yearly quota of a fishery as well as an evaluation of the impact [7]. Therefore, powerful methods for the identification of stock components and quantification of their relative proportions have to be constructed for assessing the effects of mixed-stock sea fisheries on home water stocks.

A variety of techniques have been developed for the discrimination between Atlantic salmon of North American and European origins. Early efforts include morphometric and meristic analysis [8,9,10], chemical analysis [11,12,13], protein electrophoresis methods [14], as well as nucleic acid analysis [7,15,16,17]. Techniques based on DNA analysis present advantages over protein-based techniques, as the former is not dependent on tissue source, age of the individual, or sample damage. Recently, mitochondrial DNA (mtDNA) has been sequenced and studied [18,19], and it has provided potentially useful markers for discriminating between Atlantic salmons. Restriction analysis of mtDNA has been reported to differentiate between closely related species [7,20]. However, its routine and widespread application in fishstock management is still constrained by the cost, speed, and technical complexity of existing methods.

Microfluidic methods have been developed widely for detection of biomarkers, mycotoxins and tumor cells [21,22,23]. In our hands, we have developed a microfluidic method that is based on the microfluidic microarray (MMA) assay [24,25,26]. The MMA assay consists of the hybridization of the mtDNA of Atlantic salmon with specific oligonucleotide probes. There are hundreds of copies of mtDNA, which is a small circular genome located in the mitochondria, as opposed to only two copies of genomic DNA in the cell nucleus. The specific probes used in the hybridization assay are designed based on the single nucleotide polymorphic (SNP) sites found in the non-coding genome, as described previously [27,28,29]. To develop our microfluidic microarray assay, we have examined the D-loop, which is a non-coding control region, in the salmon mtDNA and identified several SNP sites (see Table 1).

European salmon genome (nucleotides at J, K, L, M, and N are depicted in red).
3401 CAACTCTAAG TACCAGAATT TCTGACCAAA AATGATCCGG CAT**C**ACGCCG ATCAACGGAC CGAGTTACCC TAGGGATAAC AGCGCAATCC TCTCCCAGAG
…
8601 CTTCTAGAAA CAGACCATCG AATGGTTGTC CCTGTAGAAT C**T**CCAATCCG CGTCCTAGTT TCAGCTGAAG ACGTCCTTCA CTCCTGAGCC GTCCCTTCCT
…
9901 AGTCTGATTT CACTTCCACT CACTTACGCT ACTAACCATA GGAAATATTT TATTACTTCT CACCATATAT CAATGATGAC GAGACATTAT CCGAGAAGG**C**
…
10001 ACCTTCCAAG GGCACCACAC ACCTCCAGTC CAAAAAGGAC TACGCTATGG AATAATCTTA TTTATTACCT CCGAAGTATT CTTTTTCTTA GGGTTTTTCT
10101 GAGCCTTCTA CCACTCTAGT CTCTC**T**CCCA CACCTGAATT AGGAGGCTGC TGACCACCCA CAGGCATTAT TACTCTTGAC CCCTTTGAAG TACCACTTCT
…
13501 ATAACCGAGT CGG**A**GATATC GGACTTATCT TAAGCATAGC CTGATTCGCA ACAAACCTGA ACTCCTGAGA AATTCAACAA ATATTTGCCT CCTCTAAAGA

## 2. Materials and Methods

### 2.1. Materials

3-aminopropyltriethoxyl silane (APTES), 50% glutaraldehyde, sodium dodecyl sulphate (SDS) and Triton X-100 were obtained from Sigma-Aldrich. A total of 18 MΩ·cm of water was generated from an Easypure RF purification system (Dubuque, IA, USA). Photoresist (SU-8 50) and the developer solution were acquired from MicroChem Corporation (Newton, MA, USA). The Sylgard^®^ 184 silicone elastomer base and its curing agent were obtained from Dow Corning Corporation (Midland, MI, USA) to make polydimethylsiloxane (PDMS) chips. Microscope glass slides (3″ × 2″) were purchased from Fisher Scientific Company (Ottawa, ON, Canada). All other chemicals and solvents were bought from BDH Tech Inc. (Toronto, ON, Canada), and they were used without further purification.

### 2.2. DNA Primers, Probes and the Extraction of DNA

The oligonucleotides (from IDT), together with their sequences and acronyms, are listed in Table 2 for primers and Table 3 for probes.

Atlantic salmon specimens (in 300 μg fin tissues) were obtained, according to the “mouse tail tissue protocol”. DNA extractions were conducted using a DNA isolation kit (Genetra, Puregene, Qiagen, Hilden, Germany).

The salmon samples were obtained from SeaSpring Hatchery (Duncan, BC, Canada) [30]. The DNA was phenol-extracted from fin tissues according to the Qiagen protocol for isolation of total DNR from tissue [31].

Sequence of J-PCR-Eu (213 bp), shown in bold blue, is complementary to the J-Eu probe, in which the SNP site J (3444) is underlined:

5′ACAAAGCCCCCATGTGGACTGGGGGCACTGCCCCCACAACCAAGAGTCACAACTCTAAGTACCAGAATTTCTGACCAAAAAT**GATCCGGCATCACGCCGATCA**ACGGACCGAGTTACCCTAGGGATAACAGCGCAATCCTCTCCCAGAGTCCCTATCGACGAGGGGGTTTACGACCTCGATGTTGGATCAGGACATCCTAATGGTGCAGCCGC-3′.

### 2.3. PCR Amplification

PCR amplifications were performed on mtDNA (50 ng) in a 25 μL reaction tube. The PCR reagents used (in 25 μL) included DNA polymerase Taq (0.05 U), forward and reverse primers (each of 12.5 pmol), 10× PCR buffer (2.5 μL), dNTPs (12.5 μmol) and autoclaved water. The PCR cycle was: initially 4 min at 95 °C, followed by 35 cycles consisting of 95 °C for 45 s (denaturation), 45 s at the optimal annealing temperature specific to each pair of primers, 72 °C for 1 min (extension), and finally at 72 °C for 10 min. Following amplification, the PCR products (5 μL) were electrophoresed on an agarose gel (1% in 1× TBE), with product sizing verified using the 1-kb DNA ladder. After the PCR products (20 μL) were purified using the PCR product purification kit (QIAquick, Qiagen, Hilden, Germany), they were quantified using a UV spectrometer (Nanodrop 2000, Mettler Toledo, Mississauga, ON, Canada).

### 2.4. Surface Modification of Glass Chips

The glass slide surfaces were aldehyde-functionalized based on an established procedure [27]. Briefly, plain rectangular slides were cleaned with a NaOH solution (10%) for 10 min at ~100 °C. After the slides were rinsed with distilled water, they were treated with a piranha solution (conc. H_2_SO_4_ to 30% H_2_O_2_ = 70:30 *v*/*v*) for 1 h at ~80 °C. Then, the slides were washed with H_2_O and dried with N_2_ gas.

The slides were then surface-functionalized in a mixture of C_2_H_5_OH: H_2_O: APTES (95:3:2 by volume) for 2h under stirring. The amine-treated slides were rinsed with 95% C_2_H_5_OH and deionized H_2_O, and dried under N_2_ gas and heated at ~120 °C for 1 h. The aminated glass slides were then treated in glutaraldehyde (5% in 10× PBS) overnight. The aldehyde-functionalized slides were washed with acetone and deionized H_2_O. After the aldehyde-modified glass slides were dried in N_2_, they were stored in a dark place at 4 °C.

### 2.5. Fabrication of PDMS Channel Plates

A PDMS channel plate (2″ × 2″) was fabricated by photolithography as previously reported [24,27]. The channel pattern of the photomask was designed using Visual Basic (Microsoft) and was printed (at 3368 dpi) on a plastic transparency film. The molding masters were fabricated as follows: First, a Si wafer (4″ diameter) was spin-coated with a layer of SU-8 photoresist. Then the channel patterns were created on the SU-8-coated wafer with the photomask by UV exposure. The unexposed regions on the SU-8-coated wafer was developed to produce the molding master. This was used for casting by the PDMS prepolymer and cured at 50 °C for 12 h to yield the PDMS plate with microchannels (300 µm in width, 20 μm in height, and 30 mm in length). Both ends of the channels on the PDMS channel plate were punched using a flat-end syringe needle to create the solution reservoirs (1 mm in diameter).

### 2.6. Probe Line Creation

**Figure 1:**
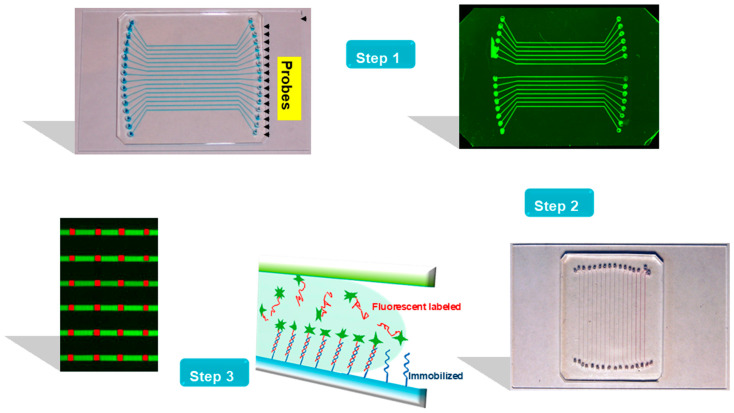
The hybridization-based assay using a microfluidic microarray (MMA) biochip with straight microchannels. (**Step 1**) A DNA probe line array on an aldehyde-modified glass slide via horizontally oriented microchannels was created. After removal of the PDMS slab, green probe lines, which were fluorescein-labeled ADF, were depicted on the glass slide, as previously reported [21]. (**Step 2**) After the straight PDMS channels were placed orthogonal to the probe lines, the MMA biochip was ready for introduction of DNA samples labeled by Cy5 (red fluorescence). (**Step 3**) Hybridization reactions between the DNA samples and the immobilized probes occurred inside the microchannels, leading to the reactions revealed at the intersections between the channels and horizontal probe lines as red patches.

The PDMS channel plate was first sealed against the glass slide to form the microfluidic biochip, as shown in Figure 1 (Step 1). Then, the DNA probes, which were prepared in a spotting solution (0.8 µL; 1.0 M of NaCl + 0.15 M of NaHCO_3_), were added into the inlet reservoirs. By using suction at the outlets, the probe solutions were filled through the channels. After the glass slides were incubated at room temperature for 30 min, covalent Schiff linkage was formed between the amine ends of the probe oligonucleotides and the aldehyde groups on the glass surface [21]. The microchannels were washed with 1 µL of a solution (0.15% Triton-X 100, 1.0M of NaCl and 0.15M of NaHCO_3_). Then, after the PDMS plate was removed, the slide was chemically reacted with a reducing solution (100 mg of NaBH_4_ dissolved in 30 mL of 1× PBS and 10 mL of 95% EtOH) for 15 min. Finally, the glass chip was rinsed with deionized water for 2 min and dried by N_2_.

### 2.7. Target DNA Hybridization and Fluorescence Scanning

DNA hybridizations occurred in the straight channels which were orthogonal to the printed probe lines on the slide (as shown in Figure 1, Step 2). The DNA target samples were prepared in a hybridization buffer (4× SSC + 0.2% SDS) as usual. Just before hybridization, the PCR products were first denatured at 95 °C for 4 min, and then they were promptly cooled in an ice-water bath. DNA targets (1 µL) were added to the inlet reservoirs on the PDMS chip, and suction was applied to pull in the solutions. The flow of DNA targets and their hybridization with the immobilized probes occurred in the microchannels, as shown in Figure 1, Step 3. The hybridization temperature was controlled either in the continuous-flow mode or the stop-flow mode. In the former mode, a thermoelectric device (CP1-12715, Thermal Enterprises, Wildwood, NJ, USA) was placed under the glass slide assembly, and the hybridization temperature was controlled by adjusting the electric voltage. In the stop-flow mode, the assembly was incubated in a humid container placed in an oven at a specified temperature. The hybridization reaction occurred in the microchannels of the size of 300 µm × 300 µm. The microchannels were washed with the hybridization buffer (2 µL) as soon as the reaction was completed.

After hybridization and washing, the glass slide was fluorescently scanned on a confocal laser scanner of a resolution of 25 µm. The excitation wavelength was 488 nm for fluorescein-labeled samples or 633 nm for Cy5-labeled samples.

## 3. Results and Discussion

### 3.1. Identification of SNP to Discriminate Between Atlantic Salmon of Different Continental Origins

In genetic studies, it is very popular to investigate the mitochondrial genome because of its ease of isolation (i.e., more easily than from the nuclear genome), its small size, and its rapid accumulation of mutations.

The salmon mitochondrial genome has been identified to have 215 SNP sites. From the DNA sequences among various salmon samples, we have selected five SNP sites (J, K, L, M, and N) at nucleotide positions 3444, 8642, 10000, 10126, and 13514, respectively (see Table 1), due to complete consensus, as shown in Table 1. The positions 2850 and 3711 with only partial consensus are also shown to illustrate the selection process. The European salmon DNA sequence at these five SNP sites is also shown below in Table 1, in which the sites are depicted in red color.

From the sequence and SNP site information, primers and probes are designed to give similar, if not identical, melting temperatures (T_m_), based on interrelated governing factors, such as GC content, nucleotide length, primer concentration, as well as concentrations of Na^+^ and Mg^2+^ ions. The forward (For) primers and reverse (Rev) primers are designed so that the T_m_ values of each primer pair are matched. Each forward primer is fluorescently labeled by Cy5 so that the forward strand of the PCR product formed is complementary to its specific probe, as shown in Table 3.

The forward strand of the European salmon PCR product around SNP site J (position 3444) is depicted below in Table 2, in which the site is underlined, and the J probe is bolded in blue color.

Complementary sequences for probes J-Am and J-Eu (biotin- and Cy5-labeled), and those for probes K-Am and K-Eu (Cy5) were also ordered.

### 3.2. Hybridization of PCR Products of Sample 1

**Figure 2:**
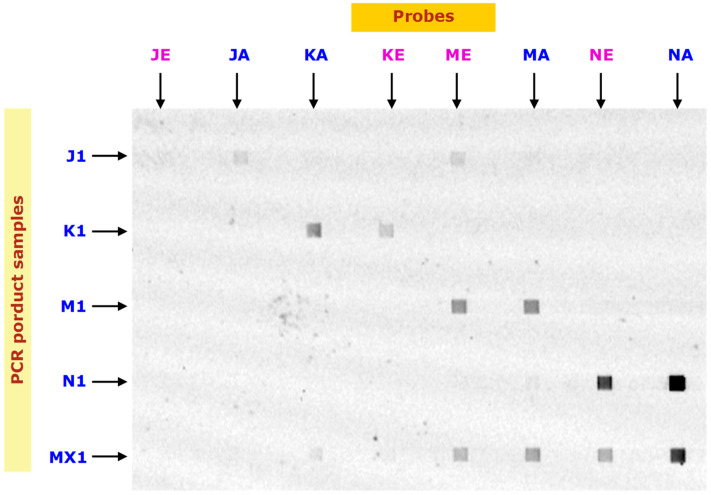
Images of the hybridizations of PCR products (introduced in horizontal directions) with immobilized probes (immobilized vertical lines). Probes (1 mL) specific to four SNP sites (J, K, M, and N; see Table 3) of American salmon (Am or A) and European salmon (Eu or E) were immobilized for 20 min. PCR products of salmon sample 1 using four primer sets (see Table 2) were prepared. J1, K1, M1 and N1 are denatured PCR products (20 nM) and MX1 is their mixture (5 nM each). Hybridization was achieved at 50 °C in 10 min. The hybridization patches are 200 mm × 200 mm. All four PCR products prepared from sample 1 were verified by Sanger sequencing (ABI Prism 3100 genetic analyzer) to be of North American origin.

**Figure 3:**
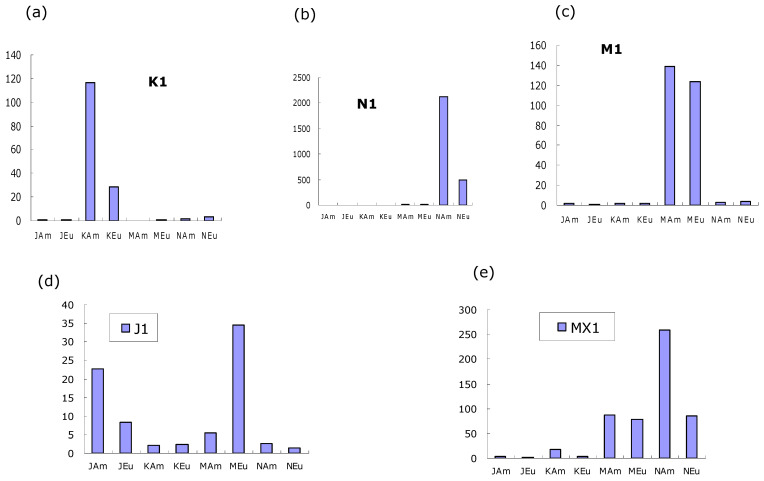
Hybridization signals between four PCR products, (**a**) J1, (**b**) K1, (**c**) M1, (**d**) N1, and (**e**) their mixture of salmon sample 1 and their respective eight probes. The signals of the binding of K1, M1 and N1 with their specific probes (KAm, MAm, and NAm) are stronger than that of J1 (with probe JAm). The discriminations of sample 1 by probes KAm and NAm are greater than that by probe MAm. Undesirably, J1 cross-hybridized with M probes in the reverse order, i.e., higher signal with MEu than with MAm. This cross-hybridization had further reduced the discrimination of the M probes in the PCR product mixture (MX1).

**Figure 4:**
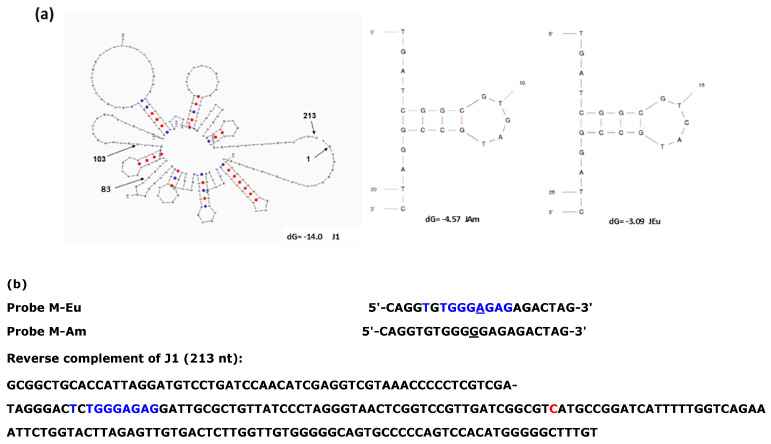
Hybridization between J probes (JAm and JEu) and J-PCR product (J1) of salmon sample 1. (**a**) Secondary structures in J1 product as well as in JAm and JEu probes. The base pairing between A and T is given by a blue dot; whereas that between G and C by a red dot. (**b**) Nucleic acid sequences of J1 around the M site and of probes MAm and MEu. The 9 nucleotides (nt) of cross-hybridization between J1 and MEu probes (21-nt) are shown in blue, with the M site shown by underlining.

Four PCR products (J1, K1, M1, and N1) and their mixture (MX1) were prepared from salmon sample 1, and hybridization of their forward strands with the probes was conducted on an MMA chip. A fluorescence image of the hybridization results is shown in Figure 2. It is observed that the hybridization signals of K1, M1 and N1 with KA, MA and NA, respectively, are strong. Since the signals are weaker in KE and NE but not ME, the discriminations were good in K1 and N1 but bad in M1. The quantified fluorescent intensities are shown in Figure 3, indicating strong and discriminative binding of K1 to KAm and N1 to NAm. The signal of M1 to MAm is strong but not much stronger than that to MEu. The signals of hybridizations between J probes and J1 were weak (note the scale in Figure 3d). The weak J signal is due to weak binding because of the secondary structures in J1 as well as hairpins in the J probes (JAm and JEu); see Figure 4a.

Even worse, J1 is found to cross-hybridize to M probes as well, as depicted in Figure 3d, in which probes MAm and MEu bind to M1. The cross-hybridization was in the reverse order, i.e., J1 binds to MEu more strongly, as shown in the alignment of TGGGAGAG between MEu (but not MAm) and the reverse complement of J1 (Figure 4b). This cross-hybridization reduced the discrimination by the M probes in the PCR product mixture MX1, as shown in Figure 3e.

We found that the signal intensity and specificity are good only for PCR products K1 and N1. With this information, sample 1 was identified to be of North American origin, and its DNA sequence was confirmed by Sanger sequencing.

### 3.3. Hybridization of Multiplex PCR Products of Three Salmon Samples

The signal intensity and specificity of hybridization between the probes and PCR products worked well with probes K and N. Since the J probes produced weak signals and the M probes were non-discriminating, we decided to select a fifth SNP site, which is L at position 10000; see the SNP nucleotides, primers and probes in Table 1, Table 2 and Table 3, respectively.

In addition, we performed multiplex PCR for J, K, L and N using the four sets of forward and reverse primers. We also conducted an analysis to ensure there was no competitive binding between the primers which were mixed in the multiplex PCR procedure. We performed multiplex PCR on three Atlantic salmon samples (1, 2, and 3).

**Figure 5:**
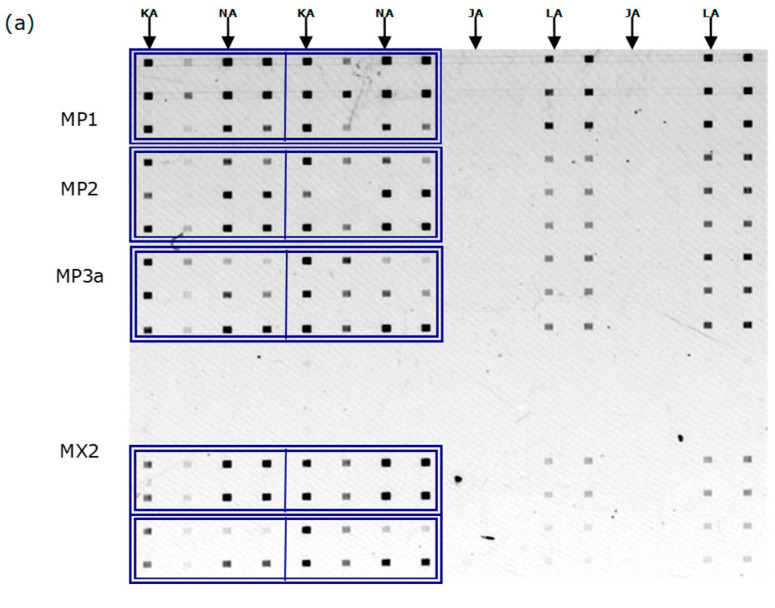

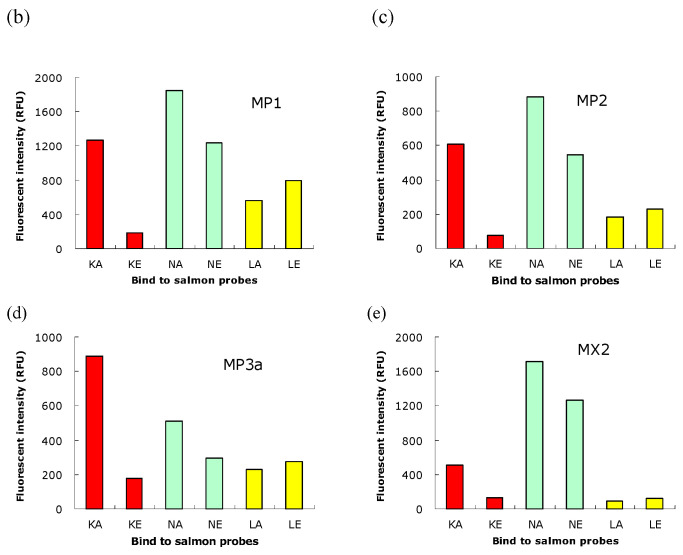
Hybridization signals of PCR products of the salmon samples (in triplicates for MP1, MP2 and MP3a and in quadruplicates for MX2) to specific probes: J, K, L, and N (in duplicates). (**a**) Fluorescence image of the hybridization assay conducted on the MMA biochip. The hybridization patches are 200 mm × 300 mm. The PCR products, MP1, MP2, MP3a (multiplex, n = 3) and MX2 (mixture of K2, N2, L2 and J2, n = 4), were introduced via the horizontally oriented microchannels. The probes lines were immobilized in the vertical orientation (in duplicates); the probes specific to the North American salmon are KA, NA, LA, and JA, while the unlabeled probes on the right are the corresponding European probes: KE, NE, LE, and JE. Duplications of PCR products hybridized with probes K and N are boxed, and duplications of probe lines are duly labeled. (**b**) Fluorescence intensity of binding of MP1 with 3 probes (K, L, and N). Intensity with probe J was too weak to show in the same scale. (**c**) Fluorescence intensity of binding of MP2 with probes K, L, and N. (**d**) Fluorescence intensity of binding of MP3a with probes K, L, and N. (**e**) Fluorescence intensity of binding of MX2 with probes K, L, and N.

The hybridization signals of multiplex PCR products of the 3 samples (MP1, MP2 and MP3a) are shown in Figure 5. MX2 was the mixture of individual PCR products of sample 2. We found that probes K and N were good at achieving discrimination, though not the L probes, see Figure 5b. This concludes that the 3 samples were of North American origin. These findings were confirmed with Sanger sequencing. Although probe L did not work well in terms of specificity, we still have probes K and N at our disposal.

### 3.4. Thermodynamic Calculations of Hybridizations for Specific Probes

Finally, we calculated the thermodynamic parameters for the five probes and attempted to predict why M and L are non-discriminating. Table 4 depicts the nucleotides involved in the mismatch duplex formation, with the discrimination ratios calculated. The free energies of perfect-matched (PM) and mismatched (MM) duplexes of the five probes and the differences in the two free energies were calculated (see Table 5).

**Figure 6:**
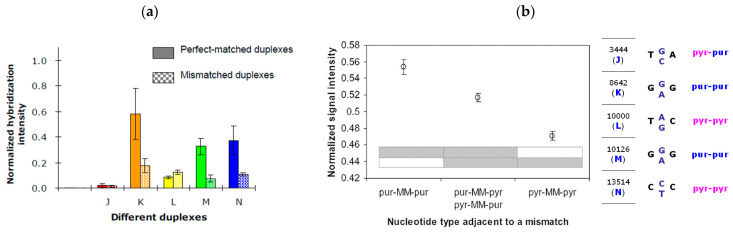
Predicted hybridization signals of perfect-matched (PM) and mismatched (MM) DNA duplexes. (**a**) PM vs. MM signals for hybridization of the PCR product with five salmon probes J, K, L, M, N. (**b**) predicted MM signals for the five probes with a consideration of the two adjacent nucleotides; T and C are pyrimidines (pyr in pink), and A and G are purines (pur in blue).

Figure 6 shows the simulated hybridization intensity of the 5 sets of probes for J, K, L, M, and N. High hybridization intensities and good discrimination are apparent in the cases of K and N (see Figure 6a). In the case of L, discrimination is undesirable due to thermodynamic reasons. However, discrimination was predicted to be good in the case of M due to thermodynamics, but the reality is not like this because kinetics and secondary structures have not been accounted for.

Figure 6b illustrates the situation when the bases (purine or pyrimidine) of the nucleotides adjacent to the SNP sites are considered.

These thermodynamic considerations have confirmed that the discrimination is achievable only by K and N, but not J, M and L.

## 4. Conclusions

In summary, we have developed a hybridization-based method to allow for the identification of the origin of the Atlantic salmon based on two SNP sites. Such a method, after proper validation, may be cost-effective, rapid, and relatively simple. In addressing the issue of cost-effectiveness, the cost of the hybridization assay is lower than previous methods, as no restriction enzymes are required.

## Figures and Tables

**Table 1:** Selected SNP sites (J, K, L, M, and N) in mtDNA of Atlantic salmon are shown with the nucleotides (C, T, A, G) and their positions (from 2850 to 13514). Partial sequences of the European salmon genome are shown below.

	European										North American			
Position	K1	K31	K28	Swed	Mork1	K42	K36	WGreen	LoirV	Alm	Nfld	UngBay	ConRiv	NB
2850	G	G	G	G	A	G	G	G	A	G	A	A	A	A
3444 (J)	C	C	C	C	C	C	C	C	C	C	G	G	G	G
3711	T	T	T	T	T	T	T	T	T	T	C	C	C	T
8642 (K)	T	T	T	T	T	T	T	T	T	T	C	C	C	C
10126 (M)	T	T	T	T	T	T	T	T	T	T	C	C	C	C
13514 (N)	A	A	A	A	A	A	A	A	A	A	G	G	G	G
10000 (L)	C	C	C	C	C	C	C	C	C	C	T	T	T	T

**Table 2:** Oligonucleotide primer sets for preparation of PCR products. For notations of J, K, L, M, N, see Table 1. The melting temperature, T_m_, was determined by us as well as by the IDT website (200 nM of primer concentration, 50 mM of monocation, and 0.7 mM of Mg^2+^) http://arep.med.harvard.edu/cgi-bin/adnan/tm.pl (accessed on 1 January 2026).

Acronym	Length	Sequence (5′-3′)	T_m_ by Lin	T_m_ IDT
J	21 21	For: Cy5—ACAAA GCCCC CATGT GGACT G Rev: GCGGC TGCAC CATTA GGATG T	75 75	60.2 60.3
K	21 19	For: Cy5—TATGA ATACA CCGAC TACGA A Rev: CTGTT TGGTT TAATC GTCC	61.9 61.6	50.5 48.8
L	19 21	For: Cy5—TGCCC TTCTA CTTAC ATCA Rev: AAAGA ATACT TCGGA GGTAA T	60.1 60.0	50.2 48.7
M	21 18	For: Cy5—CAAAA AGGAC TACGC TATGG A Rev: TGTGG TGGGC TCATG TAA	65.5 65.4	52.4 53.7
N	21 18	For: Cy5—TGAGA AGGTG TTGGC ATTAT A Rev: GGGAG TGTGA GGTCG AGT	64.3 64.4	51.4 56.3
J-PCR	213-bp	Sequence shown below		
K-PCR	226-bp			
L-PCR	215-bp			
M-PCR	223-bp			
N-PCR	209-bp			

T_m_ values, determined from IDT, were based on 50 mM of NaCl.

**Table 3:** Oligonucleotide probes used in this study (50 mM of monocation and 0.7 mM of Mg^2+^) and determined from http://arep.med.harvard.edu/cgi-bin/adnan/tm.pl (accessed on 1 January 2026). For notations of J, K, L, M, N, see Table 1.

Acronym	Length	Sequence (5′-3′)	T_m_ Lin	T_m_ IDT
J	21	Am: NH_2_-(CH_2_)_6_—TGATCGGCGTCATGCCGGATC Eu: NH_2_-(CH_2_)_6_—TGATCGGCGTGATGCCGGATC	58.4 58.4	62.1 62.1
K	21	Am: NH_2_-(CH_2_)_6_—CGCGGATTGGGGATTCTACAG Eu: NH_2_-(CH_2_)_6_—CGCGGATTGGAGATTCTACAG	54.0 51.8	57.8 55.3
L	21	Am: NH_2_-(CH_2_)_6_—CTTGGAAGGTACCTTCTCGGA Eu: NH_2_-(CH_2_)_6_—CTTGGAAGGTGCCTTCTCGGA	52.1 55.3	56.0 59.2
M	21	Am: NH_2_-(CH_2_)_6_—CAGGTGTGGGGGAGAGACTAG Eu: NH_2_-(CH_2_)_6_—CAGGTGTGGGAGAGAGACTAG	54.6 52.1	58.6 56.0
N	21	Am: NH_2_-(CH_2_)_6_—GTCCGATATCCCCGACTCGGT Eu: NH_2_-(CH_2_)_6_—GTCCGATATCTCCCGACTCGGT	56.5 54.1	60.5 57.9

T_m_ values, from IDT, were provided based on 50 mM of NaCl.

**Table 4:** Mismatch types of European (left, white) and North American (right, yellow) salmon.

	3444 (J)		8642 (K)		10000 (L)		10126 (M)		13514 (N)	
Mismatch	C/C	G/G	G/T	A/C	A/C	G/T	G/T	A/C	C/A	T/G
Discrimination ratio	0.5	0.4	0.7	0.4	0.4	0.7	0.7	0.4	0.4	0.7

**Table 5:** The calculated free energies of DNA duplexes for North American salmon PCR products created around the five SNP sites J, K, L, M, N: mismatched (MM) is with European probes and perfect-matched (PM) with North American probes *.

Duplex Name	Mismatched Part for MM Duplexes	ΔG of PM Duplex (kJ/mol)	ΔΔG of PM and MM Duplexes (kJ/mol) ^#^
J	TCA:ACT	−91.2	15.89
K	GGG:CTC	−76.9	7.11
L	TAC:ACG	−84.1	18.4
M	GGG:CTC	−79.5	7.11
N	CCC:GAG	−82.8	14.22

* Calculated at 50 °C and in 0.4 M of NaCl using the DINAMelt Web Server [32,33]. ^#^ ΔΔG = (ΔG of MM duplexes) − (ΔG of PM duplexes).

## Data Availability

The original contributions presented in this study are included in the article. Further inquiries can be directed to the corresponding author.

## Abbreviations

The following abbreviations are used in this manuscript:

MMA	Microfluidic microarray
PDMS	Polydimethylsiloxane
APTES	3-Aminopropyltriethoxysilane
SDS	Sodium dodecyl sulphate
SSC	Sodium chloride–sodium citrate buffer
PBS	Phosphate buffered saline
